# Treatment of three pediatric AML co-expressing NUP98-NSD1, FLT3-ITD, and WT1

**DOI:** 10.1186/s12887-024-04954-1

**Published:** 2024-07-27

**Authors:** Li Liu, Qi Nie, Zugang Xiao, Xin Chen, Chunhui Yang, Xiaoyan Mao, Na Li, Yan Zhou, Qulian Guo, Xin Tian

**Affiliations:** 1 Department of Hematology, The Affiliated Children’s Hospital of Kunming Medical University, Kunming Medical University, Kunming, China; 2 Department of Pediatrics, QuJing Medical College, Qujing, China; 3 Department of Pediatrics, Da Li University, Da Li, China; 4 Kunming Kingmed Institute for Clinical Laboratory Co., Kunming, China; 5 Department of Hematology, Kunming Children’s Hospital, Kunming, China; 6 Department of Pediatrics, Sichuan Clinical Research Center for Birth Defects, The Affiliated Hospital of Southwest Medical University, Luzhou, China

**Keywords:** Pediatric, AML, FLT3/ITD, NUP98/NSD1, WT1, Haplo-HSCT

## Abstract

During the treatment of 89 pediatric patients with Acute Myeloid Leukemia (AML) at the Hematology Department of Kunming Medical University’s Children’s Hospital from 2020 to 2023, three patients were identified to co-express the NUP98-NSD1, FLT3-ITD, and WT1 gene mutations. The bone marrow of these three patients was screened for high-risk genetic mutations using NGS and qPCR at the time of diagnosis. The treatment was administered following the China Children’s Leukemia Group (CCLG)-AML-2019 protocol. All three patients exhibited a fusion of the NUP98 exon 12 with the NSD1 exon 6 and co-expressed the FLT3-ITD and WT1 mutations; two of the patients displayed normal karyotypes, while one presented chromosomal abnormalities. During the induction phase of the CCLG-AML-2019 treatment protocol, the DAH (Daunorubicin, Cytarabine, and Homoharringtonine) and IAH (Idarubicin, Cytarabine, and Homoharringtonine) regimens, in conjunction with targeted drug therapy, did not achieve remission. Subsequently, the patients were shifted to the relapsed/refractory chemotherapy regimen C + HAG (Cladribine, Homoharringtonine, Cytarabine, and G-CSF) for two cycles, which also failed to induce remission. One patient underwent Haploidentical Hematopoietic Stem Cell Transplantation (Haplo-HSCT) and achieved complete molecular remission during a 12-month follow-up period. Regrettably, the other two patients, who did not receive transplantation, passed away. The therapeutic conclusion is that pediatric AML patients with the aforementioned co-expression do not respond to chemotherapy. Non-remission transplantation, supplemented with tailor-made pre- and post-transplant strategies, may enhance treatment outcomes.

**Supplementary Information:**

The online version contains supplementary material available at 10.1186/s12887-024-04954-1.

## Introduction

In pediatric acute myeloid leukemia (AML), co-expression of NUP98-NSD1 and FLT3-ITD gene abnormalities are frequently associated with poor prognosis [1]. The NUP98-NSD1 fusion gene arises from a chromosomal translocation t (5;11) (q35; p15.5), involving the NUP98 gene on the short arm of chromosome 11 and the NSD1 gene on the long arm of chromosome 5. It represents the most prevalent rearrangement of NUP98 and was classified as a high-risk factor in AML according to the 2017 European Leukemia Net guidelines [2]. Literature reports [3] indicate that AML patients positive for NUP98/NSD1 exhibit significantly higher white blood cell counts than their NUP98-NSD1 negative counterparts, with the FAB-M4/M5 phenotype being more prevalent. The presence of FLT3-ITD (internal tandem duplication) and WT1 mutations are also more likely to occur. NUP98-NSD1 stands as an independent prognostic factor for adverse outcomes. For both pediatric and adult AML patients positive for NUP98-NSD1, the 4-year event-free survival(EFS) rate is markedly low. Initial responses to conventional chemotherapy are suboptimal in patients positive for NUP98-NSD1. The therapeutic response can be partially enhanced with Venetoclax/Azacitidine, while adverse therapeutic outcomes may be reversed with the conjunction of FLT3 inhibitors and allogeneic hematopoietic stem cell transplantation [4]. This study systematically analyzes the biological characteristics and clinical efficacy in our center’s pediatric AML patients co-expressing NUP98-NSD1, FLT3-ITD, and WT1.

## Methods

### Study population

We retrospectively analyzed 89 pediatric AML patients aged 0–14 treated at The Affiliated Children’s Hospital of Kunming Medical University from January 1, 2020, to August 20, 2023. The diagnosis of AML was based on the 2016 edition of the World Health Organization (WHO) Classification of Tumors of Hematopoietic and Lymphoid Tissues [5]. All patients were confirmed through integrated diagnosis involving cytology, immunology, cyto-genetics, and molecular biology. Inclusion criteria included a clinical diagnosis of AML and the availability of complete medical records for follow-up. This study received approval from the Ethics Committee of Kunming Children’s Hospital [Approval No: IIT2020019-EC-2], and the guardians of the patients signed an informed consent form, agreeing to the use of samples and clinical data for scientific research analysis.

### Data collection

We gathered general information from each patient and used bone marrow fluid samples preserved with EDTA anticoagulant at the time of initial diagnosis for fusion gene detection. Bone marrow nucleated cells (BMNCs) were isolated and frozen. The methodology for RNA extraction and reverse transcription was consistent with our previous research [6, 7]. Amplification primers for the NUP98-NSD1 fusion transcript were referenced from the literature [3]. Total RNA was denatured, fragmented, and reverse-transcribed to yield double-stranded DNA fragments of 300–400 bp. Following end-repair of these DNA fragments and the addition of an adenine base, specific adapters were ligated. This was followed by PCR amplification to construct a transcriptome library, which was sequenced on the Illumina platform. The output data was subsequently analyzed. Analytical standards referred to the “2016 International System for Human Cytogenetic Nomenclature“ [8]. The same methodology was employed for the detection of the FLT3-ITD fusion gene. Amplification primers for the FLT3-ITD fusion transcript were based on literature [9].

Detailed patient baseline characteristics, treatment responses, and gene mutation profiles are provided in the supplementary information files (Supplementary Tables 1–3).

### Treatment protocol

Under the CCLG-AML-2019 induction therapy protocol, the DAH (daunorubicin, cytarabine, and high-dose homoharringtonine) and IAH (idarubicin, cytarabine, and high-dose homoharringtonine) regimens were combined with sorafenib, a targeted drug for FLT3-ITD positive cases. For relapsed/refractory chemotherapy, the C + HAG regimen (cladribine, high-dose homoharringtonine, cytarabine, G-CSF) was used for two cycles. One patient underwent haploidentical hematopoietic stem cell transplantation (Haplo-HSCT). The pre-transplant conditioning regimen for this patient was mBuCy + ATG: cytarabine 4 g/m^2 on days − 10 and − 9; busulfan 9.6 mg/kg (intravenous) from day − 8 to -6; cyclophosphamide 3.6 g/m^2 on days − 5 and − 4; and rATG 10 mg/kg from day − 5 to -2.

## Results

### High-risk gene screening

**Fig. 1:**
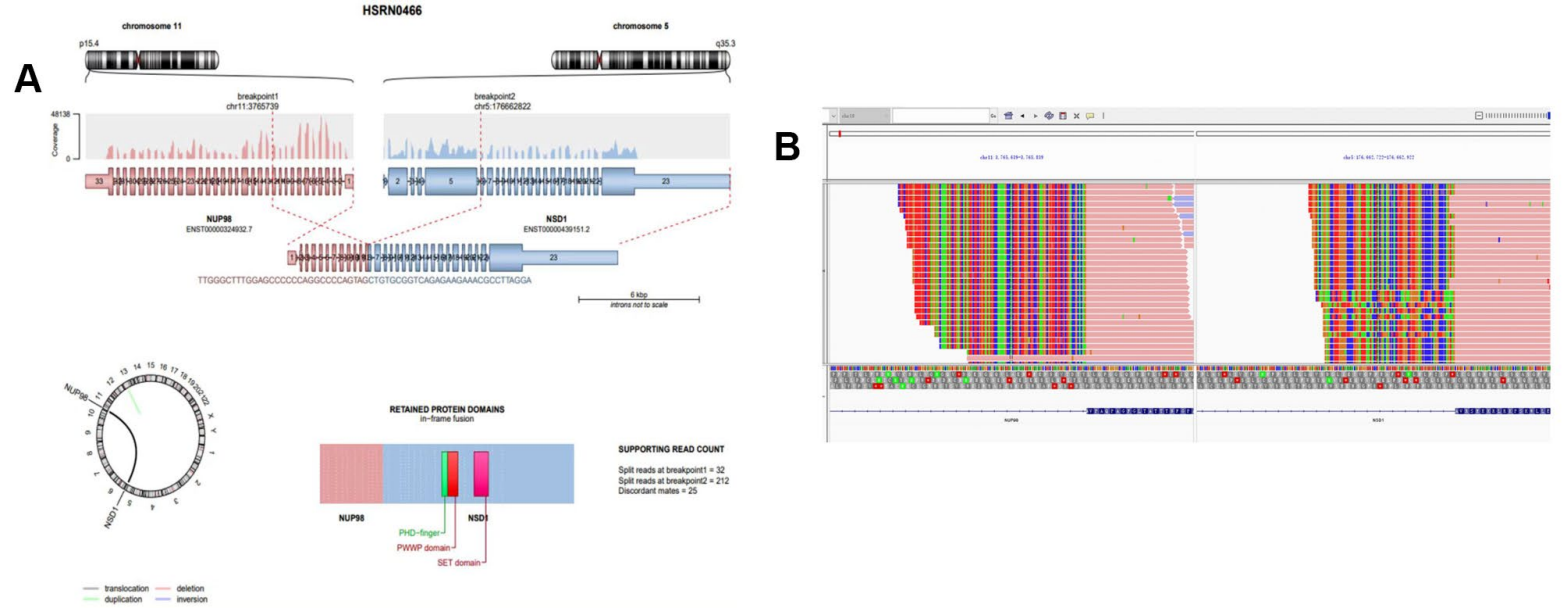
Visualization of NUP98-NSD1 fusion gene sequence data using IGV (Integrative Genomics Viewer). (**A**): The magnified view displays the junction region of the fusion gene, precisely delineating the breakpoints of the NUP98 and NSD1 genes. Peaks in the figure are differentiated by red and blue, representing the read coverage of each respective gene, thereby revealing the exact location of the fusion gene. Gene annotation tracks illustrate the positions of NUP98 and NSD1 on their respective chromosomes, as well as their exon and intron structures. Sequence details near the fusion point are highlighted to reveal the molecular mechanism of the fusion event. The circular diagram below illustrates the types of chromosomal variations involved in the fusion event between NUP98 and NSD1 genes, with green indicating translocation events, blue representing deletions, and purple signifying inversions. (**B**): The bar graph in the figure provides a detailed description of the protein domains retained in the fusion protein of NUP98 and NSD1. Different colored blocks represent specific functional domains, such as the PHD finger domain (green), PWWP domain (red), and SET domain (blue). The supporting read counts section presents the number of sequence reads validating the gene fusion points, where ‘split reads’ refer to the number of sequence reads identified at the two breakpoints, and ‘discordant pair reads’ refer to the number of paired sequence reads predicting the gene fusion

In the high-throughput sequencing (NGS) screening of 89 pediatric AML patients, we identified a variety of gene fusion and mutation combinations. The results revealed that 4 patients (accounting for 4.49%) simultaneously exhibited positive signals for NUP98-NSD1 and FLT3-ITD fusions. In these patients, fusions were observed between exon 12 of NUP98 and exon 6 of NSD1(Fig. 1A-B near here), as well as between exon 14 of FLT3 and exon 15 of ITD. Moreover, within these 4 samples, 3 patients (representing 3.37%) tested positive for the WT1 gene. Additionally, 1 patient (1.1%) exclusively showed simultaneous positivity for FLT3-ITD and WT1. Another 6 patients (6.74%) presented solely with WT1 gene mutations, accompanied by positivity for other genes. Among these 3 samples exhibiting concurrent positivity for NUP98-NSD1, FLT3-ITD, and WT1, one patient demonstrated positive results for JAK2 (p.L316_Y317insFT), OBSCN (p.R968W), SETB-P1 (p.G1067S), SP140 (p.P186R), and SUSD2 (p.P67L).The other two patients were respectively positive for AKT1S1(p.R51G), DDX41 (p.S154R), RYR1(p.E3689dup), UBR4(p.T2677I), BLM(p.K812Q), RB1(p.S82L), TYK2(p.R221W) and EPCAM(p.Q204H), GSKIP(p.D61G), KMT2D(p.E1224_S 1229del), NOTCH3(p.G131S), RPS6KA1(p.R245H). The allelic ratios (AR) of FLT3-ITD in these three pediatric patients were as follows: 0.796, 0.401, and 0.041, respectively.

### General conditions and morphological results of NUP98-NSD1, FLT3-ITD, and WTI positive patients

Among the three patients who were positive for NUP98-NSD1, FLT3-ITD, and WTI, two were females and one was male, aged 13, 6, and 8 respectively. All patients presented to the hospital after their initial diagnosis. One patient underwent Haplo-HSCT treatment. The peripheral blood white cell counts at the time of their initial diagnosis were 186.61 × 10^9/L, 61.36 × 10^9/L, and 241.71 × 10^9/L, respectively. Based on the French-American-British (FAB) leukemia morphological classification, all patients were categorized under the AML M5 subtype. Flow cytometry revealed that all cases had an AML phenotype (Table 1 near here).

**Table 1:** Morphological and genetic examination results of NUP98-NSD1, FLT3-ITD, and WT1 positive AML patients

Patient ID	Gender	Age (years)	FAB classification	Chromosomal karyotype	WT1 (gene)	FLT3(gene)
1	Female	13	AML-M5	Initial diagnosis: 46,XX;	Mutated	Positive
2	Female	6	AML-M5	Initial diagnosis: 46,XX;	Mutated	Positive
3	Male	8	AML-M5	Initial diagnosis: 46,XY, inv(5)(q15q35);	Mutated	Positive
			No remission after treatment: 46,XY, inv(5)(q15q35)		Mutated	Positive

*Note*: AML stands for Acute Myeloid Leukemia. The FAB classification refers to the French-American-British classification system that categorizes blood malignancies based on hematopoietic cell morphology

### Genetic characterization of NUP98-NSD1, FLT3-ITD, and WT1 positive patients

At initial diagnosis, all three patients had chromosomal karyotype results, with none displaying complex karyotypic abnormalities. Two patients exhibited a normal karyotype, while one patient presented with a heterozygous deletion of chromosome 13 with a normal copy number and an inversion on the arm of chromosome 5.

### Treatment and efficacy

All three patients underwent DAH and IAH treatment, yet none achieved complete remission in the bone marrow, with pathologic cytology showing more than 5% blasts. The measurable residual disease (MRD) ratios of primitive/immature cells were 1.5%, 1.8%, and 2.3%, indicating that they did not reach complete remission. Subsequent treatment shifted to the relapsed/refractory chemotherapy protocol C + HAG. Reassessment of bone marrow MRD showed 2.3%, 2.5%, and 8.1%, respectively, demonstrating persistent non-remission and poor response to chemotherapy. One patient, under the burden of tumor load, underwent salvage Haplo-HSCT. Two months later, MRD assessment revealed the absence of phenotypic anomalies in primitive/immature cells, and complete molecular remission was achieved during a 12-month follow-up. The other two patients, who did not undergo transplantation, failed to achieve remission after multiple chemotherapies and succumbed to post-chemotherapy infections (refer to Table 2 nearby).

For the child with a FLT3-ITD AR of 0.796, no negative conversion was observed after six months of combined sorafenib and intensive therapy. The patient with an AR of 0.401 turned negative one month post-transplantation, while the patient with an AR of 0.041 achieved negative conversion one month after combined sorafenib and chemotherapy.

**Table 2:** Clinical treatment outcomes of NUP98-NSD1, FLT3-ITD, and WT1 positive AML patients

Patient ID	Gender	Age (years)	Chemotherapy and targeted therapy outcomes	HSCT outcomes	OS time (months)
1	Female	13	Sequential administration of DAH, IAH, and C+HAG chemotherapy regimens combined with targeted therapies such as sorafenib, dasatinib, venetoclax, was given; however, there was a persistent lack of remission	Not transplanted	11
2	Female	6	Treatment regimen same as above	Not transplanted	15
3	Male	8	Treatment regimen same as above	12 months post salvage transplantation therapy	26

*Note*: AML: Acute Myeloid Leukemia; DAH: a chemotherapy regimen combining daunorubicin, cytarabine, and homoharringtonine; IAH: a chemotherapy regimen integrating idarubicin, cytarabine, and homoharringtonine; C + HAG: a treatment method combining cladribine, homoharringtonine, cytarabine, and granulocyte colony-stimulating factor

## Discussion

In pediatric AML, rearrangements of the NUP98 gene are relatively rare, accounting for about 3.8%. Rearrangements of the NUP98 gene are associated with a high malignancy in pediatric AML and poor treatment response [10]. NUP98-NSD1 positive AML often coexists with additional mutations in genes such as NRAS, FLT3, WT1, and MYC [11]. The FLT3-ITD (Internal Tandem Duplication) mutation is a type of FLT3 gene mutation leading to the persistent activation of the FLT3 tyrosine kinase receptor, promoting cell proliferation and inhibiting apoptosis.

Mutations in NUP98-NSD1, FLT3-ITD, and WT1 are associated with high malignancy and poor therapeutic response in pediatric AML. When these three genetic mutations coexist in pediatric AML patients, the malignancy of the disease may further increase, and the therapeutic response could be even worse. Interactions may exist between these mutations, collectively promoting disease progression. In mouse models, the combination of NUP98 fusion and FLT3-ITD alterations leads to a more aggressive form of leukemia with a shorter latency period. Consistent with this, the coexistence of NUP98 fusion and FLT3-ITD mutations can predict a poorer prognosis in AML patient populations [1]. The WT1 gene mutation, FLT3-ITD, and NUP98-NSD1 fusion gene, in varying combinations, define a subgroup of pediatric AML with a poor prognosis [12]. Therefore, for these high-risk patients, more aggressive and individualized treatment strategies are required.

This study represents the first exploration of treatment strategies for pediatric AML patients expressing a triple gene mutation (NUP98-NSD1, FLT3-ITD, and WT1). It also investigates the efficacy of a novel relapsed/refractory chemotherapy regimen, C + HAG (Cladribine, Homoharringtonine, Cytarabine, and G-CSF), combined with haploidentical hematopoietic stem cell transplantation (Haplo-HSCT). Previous research has primarily focused on the treatment of single or at most dual high-risk gene mutations. Our study expands the understanding in this field, particularly in the treatment of patient groups harboring these three specific mutations.

For AML patients positive for both NUP98-NSD1 and FLT3-ITD fusion genes and the WT1 gene, the risk of relapse is higher, and the survival prognosis is poorer. Our study results are consistent with this observation [1].

However, among these three patients, one received a haploidentical hematopoietic stem cell transplant (Haplo-HSCT) from their father and achieved molecular remission within 12 months post-transplant. This suggests that HSCT may offer a potential treatment option for AML patients with this specific genetic fusion combination.

According to the CCLG-AML-2019 protocol, NUP98 rearrangement is classified as high-risk. Even after achieving hematologic complete remission (CR), AML with high-risk NUP98 rearrangement has a high relapse rate. It is preferable to proceed immediately to transplant therapy, utilizing cord blood and HLA-mismatched unrelated bone marrow transplantation (BMT). At the very least, after achieving hematologic CR with the initial two cycles of induction chemotherapy, intensive therapy with high-dose cytarabine is still required.

An allelic ratio (AR) ≥ 0.4 of FLT3-ITD has been identified as a prognostic determinant in patients with the triple mutation (NUP98-NSD1, FLT3-ITD, and WT1) [12]. The child with an AR of 0.796 did not achieve negative conversion after treatment with the FLT3-ITD targeted drug sorafenib combined with intensive therapy.

## Conclusion

In this study, we observed that pediatric patients with acute myeloid leukemia (AML) exhibiting co-expression of NUP98-NSD1, FLT3-ITD, and WT1 in our cohort showed a lower response rate to chemotherapy. This finding indicates that transplantation in a non-remission state, combined with pre-transplant cladribine treatment strategies, may enhance treatment outcomes and prognosis for these patients.

According to the CCLG-AML-2019 protocol, NUP98 rearrangement categorizes patients into a high-risk group. Even after achieving hematological complete remission (CR), intensive chemotherapy with high-dose cytarabine is still required.

All three patients with the triple mutation and FLT3-ITD allelic ratios (AR) ≥ 0.4 did not achieve negative conversion, despite treatment with sorafenib combined with intensive therapy. The efficacy of sorafenib as a targeted therapy for FLT3-ITD positive cases is insufficient, and most current protocols favor the use of gilteritinib.

Additionally, more prospective clinical trials and long-term follow-up studies are necessary to validate the potential value of non-remission transplantation combined with personalized strategies for enhancing long-term survival and quality of life for pediatric AML patients. We anticipate that with continual advancements in this area of research, more precise and individualized treatment options will become available for this unique patient population, thereby effectively elevating their cure rates and quality of life.

### Electronic supplementary material

Below is the link to the electronic supplementary material.

Supplementary Material 1

## Data Availability

Data is provided within the manuscript or supplementary information files.
