# Supplementary material for: Treatment of three pediatric AML co-expressing NUP98-NSD1, FLT3-ITD, and WT1
Source: BMC Pediatr. 2024 Jul 27;24:483. doi: 10.1186/s12887-024-04954-1 (PMC11282587; doi:10.1186/s12887-024-04954-1)
Supplement: Supplementary file 1 — Supplementary Material 1 [file 12887_2024_4954_MOESM1_ESM.docx]

Table S1: Baseline Characteristics and Initial Diagnosis Results of NUP98-NSD1, FLT3-ITD, and WT1 Positive Patients

| Patient ID | Gender | Age (years) | WBC Count(×10^9/L) | Hemoglobin (g/L) | FAB Classification | Chromosomal Karyotype | NUP98-NSD1 | WT1 |
| --- | --- | --- | --- | --- | --- | --- | --- | --- |
| 1 | Female | 13 | 186.61 | 73 | AML-M5 | 46,XX | Positive | Positive |
| 2 | Female | 6 | 61.36 | 80 | AML-M5 | 46,XX | Positive | Positive |
| 3 | Male | 8 | 241.71 | 65 | AML-M5 | 46,XY,inv(5)(q15q35) | Positive | Positive |

Table S2: Treatment Responses and MRD Evaluation

| Patient ID | Initial Treatment Regimen | Initial Treatment Response (MRD%) | Subsequent Treatment Regimen | Subsequent Treatment Response (MRD%) | Transplant Status | Pre-Transplant Treatment Regimen | Follow-Up Time (months) | Overall Survival (months) |
| --- | --- | --- | --- | --- | --- | --- | --- | --- |
| 1 | DAH, IAH | 1.5 | C+HAG | 2.3 | Not Transplanted | N/A | N/A | 11 |
| 2 | DAH, IAH | 1.8 | C+HAG | 2.5 | Not Transplanted | N/A | N/A | 15 |
| 3 | DAH, IAH | 2.3 | C+HAG | 8.1 | mBuCy+ATG | <10^-4 | 12 | 26 |

Table S3: Detailed Genetic Mutation Information

| Patient ID | NUP98-NSD1 | FLT3-ITD (AR) | WT1 | Other Gene Mutations |
| --- | --- | --- | --- | --- |
| 1 | Positive | 0.796 | Positive | JAK2(p.L316_Y317insFT), OBSCN(p.R968W), SETBP1(p.G1067S), SP140(p.P186R), SUSD2(p.P67L) |
| 2 | Positive | 0.401 | Positive | AKT1S1(p.R51G), DDX41(p.S154R), RYR1(p.E3689dup), UBR4(p.T2677I), BLM(p.K812Q), RB1(p.S82L), TYK2(p.R221W) |
| 3 | Positive | 0.041 | Positive | EPCAM(p.Q204H), GSKIP(p.D61G), KMT2D(p.E1224_S1229del), NOTCH3(p.G131S), RPS6KA1(p.R245H) |
